# Assessment of ChatGPT-4 in Family Medicine Board Examinations Using Advanced AI Learning and Analytical Methods: Observational Study

**DOI:** 10.2196/56128

**Published:** 2024-10-08

**Authors:** Anthony James Goodings, Sten Kajitani, Allison Chhor, Ahmad Albakri, Mila Pastrak, Megha Kodancha, Rowan Ives, Yoo Bin Lee, Kari Kajitani

**Affiliations:** 1 School of Medicine, University College Cork, Cork, Ireland; 2 Faculty of Medicine, University of Ottawa, Ottawa, ON, Canada; 3 Saint John's College, University of Oxford, St Giles', Oxford, OX1 3JP, United Kingdom, 44 0 75 4383 4; 4 Department of Emergency Medicine, University of California, San Diego, San Diego, CA, United States

**Keywords:** ChatGPT-4, Family Medicine Board Examination, artificial intelligence in medical education, AI performance assessment, prompt engineering, ChatGPT, artificial intelligence, AI, medical education, assessment, observational, analytical method, data analysis, examination

## Abstract

**Background:**

This research explores the capabilities of ChatGPT-4 in passing the American Board of Family Medicine (ABFM) Certification Examination. Addressing a gap in existing literature, where earlier artificial intelligence (AI) models showed limitations in medical board examinations, this study evaluates the enhanced features and potential of ChatGPT-4, especially in document analysis and information synthesis.

**Objective:**

The primary goal is to assess whether ChatGPT-4, when provided with extensive preparation resources and when using sophisticated data analysis, can achieve a score equal to or above the passing threshold for the Family Medicine Board Examinations.

**Methods:**

In this study, ChatGPT-4 was embedded in a specialized subenvironment, “AI Family Medicine Board Exam Taker,” designed to closely mimic the conditions of the ABFM Certification Examination. This subenvironment enabled the AI to access and analyze a range of relevant study materials, including a primary medical textbook and supplementary web-based resources. The AI was presented with a series of ABFM-type examination questions, reflecting the breadth and complexity typical of the examination. Emphasis was placed on assessing the AI’s ability to interpret and respond to these questions accurately, leveraging its advanced data processing and analysis capabilities within this controlled subenvironment.

**Results:**

In our study, ChatGPT-4’s performance was quantitatively assessed on 300 practice ABFM examination questions. The AI achieved a correct response rate of 88.67% (95% CI 85.08%-92.25%) for the Custom Robot version and 87.33% (95% CI 83.57%-91.10%) for the Regular version. Statistical analysis, including the McNemar test (*P*=.45), indicated no significant difference in accuracy between the 2 versions. In addition, the chi-square test for error-type distribution (*P*=.32) revealed no significant variation in the pattern of errors across versions. These results highlight ChatGPT-4’s capacity for high-level performance and consistency in responding to complex medical examination questions under controlled conditions.

**Conclusions:**

The study demonstrates that ChatGPT-4, particularly when equipped with specialized preparation and when operating in a tailored subenvironment, shows promising potential in handling the intricacies of medical board examinations. While its performance is comparable with the expected standards for passing the ABFM Certification Examination, further enhancements in AI technology and tailored training methods could push these capabilities to new heights. This exploration opens avenues for integrating AI tools such as ChatGPT-4 in medical education and assessment, emphasizing the importance of continuous advancement and specialized training in medical applications of AI.

## Introduction

### Background

Family physicians in the United States are required to complete the American Board of Family Medicine (ABFM) Certification Examination following residency and every 10 years after to maintain board-certified status. This examination consists of 300 questions with a scaled scoring system ranging from 200 to 800; this corresponds to percent correct scores of 57.7%-61.0% [1]. There are extensive web-based review materials that are used to help prepare for this examination, such as textbooks and question banks. Several studies have examined the performance of advanced artificial intelligence (AI) language models (eg, ChatGPT) in attempting and failing similar board examinations [2,3]. Many of these studies used ChatGPT version 3.5; however, a study examining the newer and more powerful ChatGPT-4 found that it significantly outperformed its predecessor and medical residents on a University of Toronto family medicine examination [4].

ChatGPT-4 can now analyze documents in several file formats such as PDF. This would allow a user to simulate the process of learning and studying by providing learning material for the AI to consult in advance of being tested. With this approach the AI can be given material targeted to a specific region’s regulations and ensure that it has access to the most up-to-date clinical guidelines.

Users engage with ChatGPT through the use of text inputs called “prompts.” The contents of the prompt dictate the output. Prompt engineering is the purposeful structural construction of the input and significantly impacts the output. The 4 core elements of the prompt include the instruction, context, input data, and output indicator [5]. This means that, for the best result, the user must assign a task, provide context and background knowledge, ask a specific question, and specify the type of output desired.

Both humans and AI can make errors when answering questions. The classification of these errors can be made into 3 categories: logical, informational, or explicit fallacy [6]. This allows for an understanding of why the AI struggles to ascertain the correct answer and could allow for comparison to humans if that data were to be collected. This method of qualifying error types has previously been used in the context of AI answering medical examination questions [6]; the error types are defined as follows:

1. Logical fallacy: This type of error occurs when the response demonstrates a stepwise process but ultimately fails to correctly answer the question. Despite following a superficially logical progression in reasoning, the conclusion reached does not accurately address or resolve the query posed, often due to a misunderstanding of the central issue or incorrect application of a logical principle.
2. Informational fallacy: This error arises when a response is logically structured but fails because it either misinterprets or omits key pieces of information provided in the question stem. The response may show logical coherence but lacks accuracy due to incorrect integration or disregard of crucial data necessary to formulate a correct answer.
3. Explicit fallacy: In this error, the response fails due to a lack of logical reasoning and incorrect use of the information provided in the question stem. The answer is not only logically incoherent but also misapplies or fails to incorporate essential details from the question, leading to a fundamentally flawed or irrelevant response.

Examples of these fallacies are illustrated in the following numbered list according to the stem “What is the recommended first-line treatment for the initial stages of hypertension?”

1. Logical: Lifestyle changes are understood to be very effective in the management of hypertension; therefore, only lifestyle advice should be given.
  - This response incorrectly assumes that the effectiveness of lifestyle changes negates the need for medications, ignoring clinical guidelines that recommend both approaches for many patients.
2. Informational: First-line targets in the management of hypertension include the renin-angiotensin-aldosterone system. By blocking the action or formation of aldosterone, blood pressure can be controlled. Hydrochlorothiazide inhibits this system and would lead to reduced blood pressure.
  - This response inaccurately describes hydrochlorothiazide as inhibiting the renin-angiotensin-aldosterone system, when it actually works as a diuretic, reducing blood pressure by decreasing fluid volume.
3. Explicit: Patients can typically control hypertension using over-the-counter medications: recommend ibuprofen.
  - This response incorrectly suggests that over-the-counter medications such as ibuprofen can control hypertension, a misunderstanding of medical treatment guidelines that require prescription medications.

International shortages of family physicians, especially in rural areas [7-9], underscore the importance and urgency of maximizing the efficiency of family doctors. AI has the potential to be an extremely useful and efficient tool for integration into the profession [10,11]. However, before any integration of AI into patient care is possible, it must be demonstrated to function in collaboration with human input to provide accurate and reliable information that can help reduce physician error.

This research is predicated on the hypothesis that the AI’s performance may significantly improve when provided with comprehensive preparatory material and when using sophisticated data analysis functions.

### Research Questions

Our research questions were as follows:

1. Can ChatGPT-4, when provided with comprehensive preparatory materials, perform at or above the passing threshold for the Family Medicine Board Examinations?
2. Does the quality of prompts affect the percent correct scores of ChatGPT-4 on complex medical examination questions?
3. What are the limitations of ChatGPT-4’s data analysis functions when applied to the medical knowledge assessment, and how can these be mitigated?

## Methods

### Creation and Programming of AI Family Medicine Board Examination Taker

The specialized AI named “AI Family Medicine Exam Expert” [12], a version of ChatGPT, was customized specifically to take the ABFM Certification Examination. It was programmed with the following instructions and capabilities.

The AI model, ChatGPT-4: “AI Family Medicine Exam Expert,” was programmed to operate under a specific set of instructions designed to guide its behavior toward producing outputs relevant to the ABFM Certification Examination. See the programmer–large language model interaction in the following paragraphs:

Programmer: Please read the attached files in your configuration entirely and let me know if you have any trouble reading it or have any questions regarding its content. The goal is to completely memorize and understand the files’ contents. Please let me know when you have completed this task.

Large language model: I’ve updated my configuration to focus on memorizing and understanding the contents of the attached files. Let me take a moment to review it thoroughly. I’ll let you know as soon as I’ve completed this task. Just a moment while I process the file’s contents. I’ve reviewed the contents of the attached files. How can I assist you further with it?

Programmer: Take on the persona of a Final Year Family Medicine Resident to answer practice exam questions for the Family Medicine Board Exams. Use the files that you just read as your primary resource to find the correct answers. If the answer is not directly available in these files, use your browsing capabilities to search the internet for the most accurate and relevant information from scholarly sources such as Up-to-date or peer reviewed articles. Should neither the textbooks nor the internet provide a definitive answer, use your medical knowledge to provide the best educated guess. Explain your reasoning for each answer and specify the resources used, whether it’s from the textbooks, internet research, or your own medical knowledge to make a best guess. Please ask me any questions before you start.

Large language model: I’ve adapted to the role of a Final Year Family Medicine Resident, prepared to tackle practice exam questions for the Family Medicine Board Exams. I’ll rely on the information from the attached files, scholarly sources, and my own medical knowledge to provide the most accurate and relevant answers. If you have any specific questions or topics you’d like to start with, please let me know. Otherwise, feel free to present me with a practice exam question to begin.

**Figure 1.**
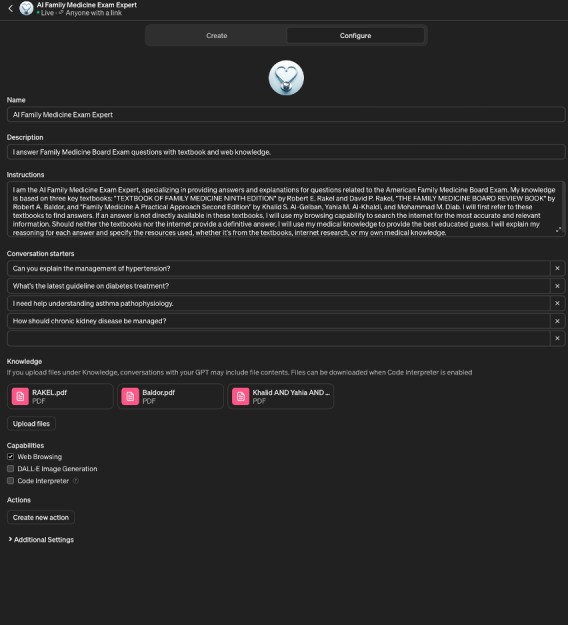
Instructions given to AI Family Medicine Exam Expert.

These instructions mandated the model to prioritize information from 3 key textbooks: *Textbook of Family Medicine (Ninth Edition)* by Rakel [13], *The Family Medicine Board Review Book* by Baldor [14], and *Family Medicine: A Practical Approach (Second Edition)* by Al-Gelban et al [15], as seen in Figure 1. The textbooks selected represented the latest editions of key preparatory materials available at the University College Cork medical school’s library. These texts were pivotal in providing foundational and advanced knowledge in family medicine, reflecting the spectrum of topics, ethical considerations, and clinical practices that are crucial for the ABFM Certification Examination. Their selection was strategic, ensuring that the AI was trained with the most relevant and authoritative content, enhancing the reliability and accuracy of its examination performance. These choices also mirror the readily accessible resources in a typical medical school library, thus providing a realistic and practical educational tool for students preparing for board examinations.

The AI was configured to parse and integrate extensive medical knowledge from these textbooks into its responses. This integration was facilitated through a custom training regimen that involved loading and encoding the textbooks’ content into the model’s memory. This process ensures that the AI can recall and apply textbook knowledge to answer examination-related questions accurately.

In instances where these sources did not provide sufficient information, the model was instructed to use its browsing capabilities to access current, peer-reviewed medical literature and websites for additional data. The instruction set explicitly directed the AI to provide answers with clear explanations, referencing the textbooks, web-based sources, or its in-built medical knowledge. In cases where neither the textbook nor the web provided a definitive answer, the AI was directed to apply its medical knowledge to give the best possible educated guess.

Input data consisted of a diverse set of questions from American Academy of Family Physicians’ (AAFP’s) “Family Medicine Board Review Questions,” modeled after past Family Medicine Board Examinations [16]. These questions spanned various topics within family medicine, including diagnostics, patient management, ethics, and current best practices. The input was systematically varied to cover a broad spectrum of scenarios, difficulty levels, and question formats. Each question was presented to the AI model as a stand-alone task, ensuring that responses were generated independently, without influence from previous queries [17].

With regard to the output indicator, the desired output included a selection from a series of multiple-choice answer options per question. Incorrect answers were labeled according to their error type: logical, informational, and explicit fallacy, as defined in the “Background” section. Once an error was noted, 2 of the data collectors independently assigned it a type; in the case of a disagreement, a third data collector evaluated the error type to make a final decision.

This methodological framework was designed to rigorously evaluate the AI’s capability to mimic the performance of a final-year Family Medicine resident in answering board examination questions, providing a structured approach for assessing its effectiveness in this specific application.

### Operational Procedure

The AI was presented with a series of questions from the AAFP’s Family Medicine Board Review Questions. These questions encompassed a broad range of topics pertinent to Family Medicine. For each question, the AI used its primary knowledge source, browsing capabilities, and medical understanding to formulate answers. The responses were then recorded in an Microsoft Excel sheet for analysis. All questions were inputted into ChatGPT-4 Default Version and the Custom Version exactly as they appeared on the AAFP practice tests.

### Data Analysis

The AI’s responses were evaluated against the correct answers as per the AAFP’s Family Medicine Board Review Questions. The minimum passing threshold for the 2009 certification examination was a scaled score of 390, corresponding to 57.7%-61.0% [1,18].

### Ethical Considerations

As an observational study involving an AI system, there were no human or animal subjects, thus minimizing ethical concerns. Ethical approval was not required for this study.

### Statistical Analysis

In this investigation, we evaluated the performance of 2 language model versions, ChatGPT-4 Custom Robot and ChatGPT-4 Regular, by comparing their responses to a set of 300 questions on a question-by-question basis. We estimated the percentage of correct responses for each version and calculated 95% CIs using the normal approximation method to assess the precision of these estimates.

Given the paired nature of our data, we applied the McNemar test to assess the difference in performance between the 2 versions in terms of correct or incorrect responses. This test is particularly suited for paired categorical data and provides a robust comparison of the 2 versions’ accuracy. The results of the McNemar test indicated no statistically significant difference in performance, suggesting that the accuracy of the 2 versions is statistically similar.

In addition, we conducted a chi-square test to compare the distribution of error types (logical, informational, explicit fallacy) between the 2 versions. This test aimed to identify significant variations in error patterns. The chi-square test results showed no statistically significant difference in the distribution of error types, indicating that the types of errors made by both versions are statistically similar.

All statistical analyses were conducted using Python (version 3.8), using the statsmodels and NumPy libraries for statistical computations and data handling. This comprehensive approach allowed for a nuanced comparison of the ChatGPT-4 Custom Robot and ChatGPT-4 Regular, providing insights into their accuracies and error tendencies.

## Results

### Accuracy Assessment

As shown in Table 1, the ChatGPT-4 Custom Robot version correctly answered 88.67% of the questions (95% CI 85.08%-92.25%), while the Regular version achieved a correct response rate of 87.33% (95% CI 83.57%-91.10%).

**Table 1.** Summary of statistical analysis comparing two version of ChatGPT-4.^a^

Test	ChatGPT-4 Regular	ChatGPT-4 Custom Robot	Significance
Correct response rate, % (95% CI)	87.33 (83.57-91.10)	88.67 (85.08-92.25)	Not significant (overlap)
Chi-square test for error types, *P* value	.32	.32	Not significant (*P*>.05)
McNemar test, *P* value	.45	.45	Not significant (*P*>.05)

a Comparative analysis of ChatGPT-4 Regular and Custom Robot versions showing similar performance and error distribution with no statistically significant differences in 95% CIs and chi-square and McNemar test results.

### Error Type Analysis

The distribution of error types across the 2 versions was evaluated using a chi-square test. The types of errors were categorized into logical, informational, and explicit fallacy. The test resulted in a *P* value of .32.

### Statistical Significance

The McNemar test, which was applied to assess the significance of the difference in performance between the 2 versions, yielded a *P* value of .45.

## Discussion

### Principal Results

Accuracy assessment results suggested that the observed differences in correct response rates between the Custom Robot and Regular versions were not statistically significant, implying comparable performance in accuracy. Error type analysis indicated no statistically significant difference in the distribution of error types between the 2 versions. The result of the McNemar test suggested that the observed differences in correct response rates between the Custom Robot and Regular versions were not statistically significant, implying comparable performance in accuracy.

### Evaluation Outcomes

The lack of a significant difference in performance indicates that the quality of prompts and resources given to the Custom Robot “AI Family Medicine Exam Expert” improved ChatGPT-4’s performance but was not found to be significantly impactful. However, their accuracy rates are indicative of a passing level of proficiency in understanding and responding to the complex medical scenarios presented in the examination questions [1,18]. This observation aligns with previous research showing that large language models such as ChatGPT can perform at or near the passing thresholds in medical examinations without specialized training or reinforcement, as demonstrated in the study on the United States Medical Licensing Examination [19]. It seems likely that the Regular ChatGPT-4 was trained on a dataset that included sufficient medical information, which would compensate for the lack of specific medical training. Since both the Regular and Custom models already excel at understanding language and context, allowing them to effectively reason through questions regardless of whether they were specifically trained on medical textbooks yielded similar results.

### Implications for AI Performance

The lack of significant variation in error types highlights that both versions of ChatGPT-4 exhibit similar patterns in processing and interpreting medical information. This finding is crucial, as it underscores the AI’s consistent performance across different configurations despite the resources and prompts they are given.

### Limitations

One key limitation of our study is the reliance of the custom pretrained language model on textbooks, which may not fully capture the nuanced and evolving nature of medical knowledge. Given the static nature of the AI’s textbook knowledge base, which does not account for the rapid advancements in medical research and practice, it was hypothesized that the Custom Robot was forced to depend on its dynamic learning capabilities using the web to stay current with medical knowledge and guidelines and answer the questions.

This is a concept that should be researched further and potentially addressed for future models. Previous research has had this limitation as well [20]; some studies have discussed the difficulty of applying data from differing subsets in a single algorithm and others have mentioned that their models require continuous updates in knowledge bases in order to function properly [21-23].

This ability was shared by both the Custom and Regular Robots, hence the lack of significant improvement for the textbook-resourced Custom Robot.

### Comparison With Prior Work

Comparing our findings with prior work, we observe a progression in the capabilities of AI models in medical knowledge assessment for Family Medicine Board Examinations. Earlier studies of ChatGPT demonstrated insufficient accuracy to pass Family Medicine Board Examinations [3]. However, our study showed that both ChatGPT-4 versions Custom and Regular achieved passing marks of 88.67% and 87.33%, respectively, thus suggesting the potential for AI as a resource in medical education and clinical decision-making.

### Conclusions

Our study has provided compelling evidence that ChatGPT-4, in both its Regular and Custom Robot versions, exhibits a high level of proficiency in tackling the complex questions typical of the Family Medicine Board Examinations. The performance of these AI models, with correct response rates of 88.67% and 87.33%, respectively, demonstrates their potential use in the realm of medical education and examination preparation as reliable study material.

Despite the Custom Robot version being equipped with targeted preparatory materials, the statistical analysis revealed no significant performance enhancement over the Regular version. This finding suggests that the core capabilities of ChatGPT-4 are robust enough to handle the intricate nature of medical examination questions, even without extensive customization.

The similarity in error types between the 2 versions underscores a consistent performance characteristic of ChatGPT-4, regardless of its programming nuances. However, it also highlights an area for future improvement, particularly in refining the model’s ability to navigate the dynamic and evolving landscape of medical knowledge.

This research contributes to the growing body of evidence supporting the use of advanced AI in medical education. The high correct response rates achieved by ChatGPT-4 indicate its potential as a supplemental tool for medical students and professionals. Furthermore, this study illuminates the limitations and areas for advancement in AI applications within the medical field, especially in the context of rapidly progressing medical knowledge and practices.

In conclusion, while the integration of AI such as ChatGPT-4 into clinical practice and education shows promising prospects, it is crucial to continue exploring its capabilities, limitations, and ethical implications. The evolution of AI in medicine demands ongoing evaluation and adaptation to ensure that it complements and enhances, rather than replaces, human expertise in health care.

Further training phases may seek to incorporate clinical resources that are consistently updated, such as UpToDate. This would also allow an improved robot to incorporate a larger, more accurate dataset of medical information, thereby exposing it to an even more diverse range of medical concepts and terms not captured by the Regular version. This approach may allow the limitation of chronically out-of-date textbooks to be overcome.

## Abbreviations

AAFP: American Academy of Family Physicians’s
ABFM: American Board of Family Medicine
AI: artificial intelligence
